# Xenobiotic Receptor-Mediated Regulation of Intestinal Barrier Function and Innate Immunity

**DOI:** 10.11131/2016/101199

**Published:** 2016

**Authors:** Harmit S. Ranhotra, Kyle L. Flannigan, Martina Brave, Subhajit Mukherjee, Dana J. Lukin, Simon A. Hirota, Sridhar Mani

**Affiliations:** 1 Departments of Medicine and Genetics, The Albert Einstein College of Medicine, 1300 Morris Park Ave, Bronx, NY 10461, USA; 2 Departments of Physiology and Pharmacology, University of Calgary, 3330 Hospital Dr. NW, Health Sciences Room 1802, Calgary, Alberta T2N4N1

**Keywords:** Orphan nuclear receptors, barrier function, intestinal innate immunity, microbial metabolites, xenobiotics

## Abstract

The molecular basis for the regulation of the intestinal barrier is a very fertile research area. A growing body of knowledge supports the targeting of various components of intestinal barrier function as means to treat a variety of diseases, including the inflammatory bowel diseases. Herein, we will summarize the current state of knowledge of *key xenobiotic receptor regulators* of barrier function, highlighting recent advances, such that the field and its future are succinctly reviewed. We posit that these receptors confer an additional dimension of host-microbe interaction in the gut, by sensing and responding to metabolites released from the symbiotic microbiota, in innate immunity and also in host drug metabolism. The scientific evidence for involvement of the receptors and its molecular basis for the control of barrier function and innate immunity regulation would serve as a rationale towards development of non-toxic probes and ligands as drugs.

## 1. Intestinal Barrier Function and IBD

The alimentary canal is a site for a large population of different microbiota (eukaryotes, archaea, bacteria and viruses) which contribute towards dietary digestion and absorption of nutrients, immunity and many other functions. The gastrointestinal tract is protected by the epithelial lining whose integrity is affected by the resident microbial metabolites through mechanisms that are mostly unclear. Compromised epithelial lining in the gut may give rise to chronic inflammation at the site. Hence, regulation of the intestinal barrier is now thought to be at the epicenter of innate immunity and homeostasis in the intestines. Dysfunction of the “intestinal barrier” is thought to play a pathognomonic role in a variety of diseases in mammalian hosts, including inflammatory bowel diseases (IBDs). A thorough understanding of the molecular regulators of the intestinal barrier is the crucial first step in not only unraveling its biology but also finding novel targets towards which new drugs can be developed. The complete biology of the intestinal barrier is complex; however, within the scope of this review article and in the context of the thematic issue, we will highlight the role nuclear receptors play in regulating intestinal barrier function.

Although the etiology of IBD, composed of Crohn’s disease (CD) and ulcerative colitis (UC) has yet to be completely elucidated, the current paradigm suggests that these diseasesare are triggered by the integration of multiple factors including specific genetic variants that govern host processes, the environment, the microbiota and immune responses [1]. The intestinal mucosa, particularly the epithelium, provides a physical barrier that prevents noxious compounds and microbes from entering the internal tissues and inappropriately activating the mucosal immune system [2, 3]. Although increased permeability is observed in patients with IBD, inflammatory processes can drive barrier dysfunction, thus it is difficult to establish causation [4]. However, data from a variety of studies suggest that compromised intestinal barrier function may contribute to the pathogenesis of IBD [5–8]. In human studies, CD patients and their first-degree relatives exhibit enhanced intestinal permeability [7, 9, 10], suggesting that a genetic contribution to barrier dysfunction may precede the manifestation of overt disease [10]. The reestablishment of intestinal barrier function may be critical to the induction and maintenance of remission in CD patients [3]. Although insufficient to cause disease on its own [11], barrier dysfunction is associated with relapse and recurrence in IBD patients [12, 13]. Thus, therapies designed to reestablish or preserve barrier function by enhancing mucosal healing and/or intestinal epithelial cell (IEC) survival, either by direct modulation of IEC function or by damping of mucosal immune responses, would prove beneficial in the treatment of IBD.

### 1.1. Host-microbe interactions in the gut

Interaction between resident microbiota and the gut shapes immunity which is helpful in maintaining host-microbe tolerance. Under normal conditions, the intestinal mucosa is maintained in a state of balance, responding to the luminal environment by activating intracellular signaling processes in the intestinal epithelium in order to maintain barrier function. Homeostatic stimulation of the intestinal epithelium provides the necessary input to activate signaling processes that contribute to a functional mucosal barrier [14]. In addition to the capacity of the innate immune system and its associated receptors to detect and initiate responses to microbial products, conserved xenobiotic and endobiotic sensing mechanisms exist in the intestinal mucosa to sense metabolic products and exogenous compounds within the lumen [15]. These systems are thought to add an additional level of defense in the gastrointestinal tract of multicellular organisms [16]. For example, it was recently reported that *C. elegans* utilizes xenobiotic sensing to initiate avoidance behaviors in the presence of pathogens and/or specific pathogenic factors [17, 18]. These responses are mediated by a family of nuclear receptors that exhibit functional similarities to the mammalian pregnane X receptor (PXR), a ligand activated nuclear receptor that plays a key role in sensing intestinal microbial metabolites [17].

### 1.2. The PXR - a xenobiotic sensor

The PXR is a member of the nuclear receptor superfamily, which includes such members as the peroxisome proliferator-activated receptor-γ (PPARγ), vitamin D receptor (VDR), glucocorticoid receptor (GR), retinoid X receptor (RXR) and many others [19]. The PXR, which is highly expressed in the liver and in IECs, is best characterized for its ability to regulate the expression of enzymes involved in drug metabolism, detoxification and excretion [20]. The PXR is also expressed in a variety of immune cells, including T cells, macrophages and dendritic cells (DC) [21], and its signaling has been reported to modulate their function through mechanisms that are poorly understood [22–25]. As a ligand-activated receptor, the PXR’s flexible binding domain allows a variety of receptor-ligand interactions to occur [26–31] resulting in its activation and translocation to the nucleus, where it forms a heterodimer with the RXR. The PXR/RXR heterodimer drives the rapid induction of gene expression through binding to genes that bear promoters containing xenobiotic-responsive enhancer modules (XREMs), including those responsible for phase I, II and III metabolic processes [32]. The PXR/RXR heterodimer can also repress gene expression through direct interactions with other transcription factor complexes [33, 34]. Thus, the current paradigm suggests that the PXR acts as part of a rapid response mechanism to enhance processes that aid in metabolism and excretion during exposure to potentially harmful xenobiotic/endobiotic compounds from environmental sources.

### 1.3. The PXR - a regulator of gut inflammation

Beyond its capacity to regulate drug metabolism, the PXR may play a more expansive role in regulating intestinal mucosal homeostasis. The biology of the PXR has been well studied in the liver, but much less is known about its role in the cells of the intestinal mucosa, including how the PXR regulates signaling pathways in IECs. Some have suggested that the PXR regulates similar cellular processes in IECs and hepatocytes, initiating the transcription of genes related to metabolism and detoxification [35]. Recent studies have implicated the PXR in the regulation of additional processes in non-IEC systems. Beyond regulating gene transcription and detoxification processes, the PXR has recently been linked to the regulation of cell migration [36], cell survival [37, 38] apoptosis[37–39] and autophagy [39]. These effects have been attributed to the PXR’s interaction with p38 MAPK [36], JNK1/2 [40, 41]and AMPK [42]. Interestingly, each of these signaling cascades regulates key functions of IECs that contribute to intestinal mucosal barrier function. We recently reported that activation of the PXR enhanced intestinal epithelial cell migration and wound healing, through the activation of a p38 MAPK dependent process [43].

In addition to aforementioned signaling cascades, the PXR can negatively regulate inflammatory signaling through its ability to inhibit NFκB [34, 44, 45]. While characterizing the reciprocal interaction between the PXR and NFκB, Zhou *et al*. reported that *Pxr*-deficient mice exhibited spontaneous small intestinal inflammation, suggesting that basal PXR activity may be required for proper mucosal health [34]. Using animal models of IBD, a number of groups have reported that the selective activation of the PXR attenuates colonic inflammation and tissue damage [43, 45–47]. Furthermore, others have reported that the stimulation of the PXR attenuates inflammation-induced barrier dysfunction and accelerates mucosal healing following a bout of colitis [43, 48]. Activation of the human PXR has also been examined using human intestinal epithelial cell lines and freshly isolated intestinal mucosal biopsies [35, 43–45]. Stimulation of the human PXR with rifaximin enhanced expression of a variety of PXR-regulated genes, including CYP3A4 and MDR1 [35, 44], and attenuated NFκB -dependent cytokine production, an effect that was lost when the PXR was knocked down using an siRNA approach [44, 45].

Taken together, it is apparent that the PXR regulates a multitude of pathways in the intestinal epithelium that contribute to the maintenance of intestinal mucosal homeostasis.

### 1.4. The PXR - a host sensor of microbial metabolites

**Figure 1:**
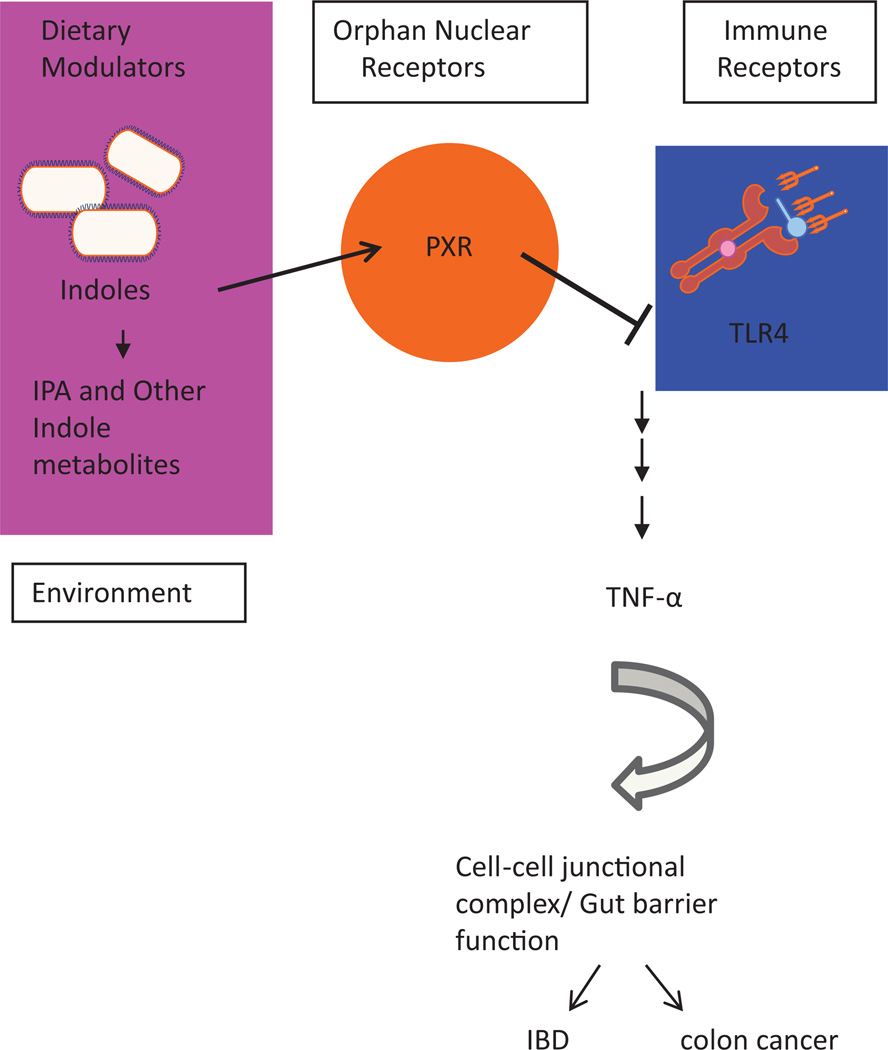
We demonstrate that in the intestines, where PXR is expressed in intestinal epithelial cells in a crypt-villus gradient (apical IEC), in homeostasis, dietary tryptophan-derived bacterial metabolites (i.e. indole-3-propionic acid or IPA) tonically activate PXR and induce a down-regulation of TLR4, and its downstream signaling pathway. This results in modulating the abundance of TNF-α, which in turn modulates intestinal barrier function (i.e. permeability). In the context of preserved indole concentrations, loss of PXR (often observed in some Crohn’s ileitis and UC) will promote the inflammatory response. In corollary, with excess loss of dietary modulators (e.g., tryptophan/IPA), and/or specific indole metabolizing bacteria (e.g., antibiotics), there is increased permeability exacerbating underlying inflammatory pathology. In this model, restitution of signaling homeostasis, either by reconstituting intestinal loss of PXR or indole-metabolite producing bacteria and/or PXR activating bacterial metabolites (i.e. IPA via tryptophan catabolism), could result in abrogating pro-inflammatory signals and loss of barrier permeability in the context of intestinal inflammation.

Despite our growing understanding of the importance of the PXR’s function in the intestinal mucosa, considerably less is known about its endogenous ligands within the GI tract. Recently, we identified a novel mode of regulation of intestinal innate immunity by intestinal microbial metabolites. We demonstrated that bacterial tryptophan metabolites (e.g., indole propionic acid) control intestinal barrier function through host cellular pathways involving the PXR and Toll-like receptor (TLR-4) [48]. Based on the experimental evidence presented in mice and human cells, we developed a model that best describes this regulatory pathway (Figure 1). To better understand the general implications, however, additional discussions of ancillary human and rodent studies are presented to buttress the foundations of our model.

Tryptophan catabolism via specific bacterial strains (e.g., indole positive *Clostridium sporogenes*) results in the production of indoles. This has been demonstrated in mice treated with clindamycin, in which, enteric bacterial metabolites of tryptophan (but not host metabolites) decreased compared to untreated mice. Notably, host metabolism of tryptophan actually increased in mice treated with clindamycin [49]. The metabolite levels returned to those of conventional untreated mice after cessation of clindamycin. Similar results have been seen in germ-free versus conventional mice [50]. Note, closely related strains are inefficient at indole metabolism. Specific strains and mammalian host tissues absorb indoles and further modify them into secondary metabolites. The metabolites expressed by host and bacteria differ chemically and likely in function [51]. The age-old paradigm that bacterial metabolites, specifically, indoles as being generally toxic compounds, are not supported by the evidence as they are naturally present in micro-to millimolar amounts in humans [52]. Bacterial targets for indole remain elusive [53]. Bacteria specific indole metabolites include indole-3-propionic acid (IPA), among others. The indoles participate in bacterial inter-kingdom signaling. Its role is central in inhibiting motility, biofilm formation, cell adherence/attachment and chemotaxis. Since these functions are altered in pathophysiologic states such as IBD, it is conjectured that in this disease there may be a loss of indole-mediated homeostasis [54]. First, indoles and in particular indole propionic acid is present in both serum and cerebro-spinal fluid (CSF) in humans [55, 56]. In a population study of the IPA concentrations observed in blood at steady-state, one study showed median concentrations ∼ 0.481 (range: 0.291–1.095) µM [57]. In another study fecal indoles were present at concentrations ∼ 1µg/g of tissue (at approximately 1/5^*th*^ of the concentration of butyrate) [58]. In another study, intestinal indole concentrations can vary from 250 µM to approximately 1 mM, depending partly on diet [59]. The average concentration range of total IPA (bound and free) was ∼ 140–183 ng/ml, with the lower range of values present in diabetics (*n* = 140) and pregnant (*n* = 20) women as compared with “normal” control subjects (*n* = 31). The results showed substantial inter-individual variation (up to 10–20 fold) that is significantly reduced by serial measurements performed repeatedly over 4 weeks. The variability in the plasma levels of IPA were attributed to differences in the intestinal flora and dietary tryptophan intake [55]. The CSF content of IPA is much less and estimated to be in the range of 300:1 (plasma:CSF) [60]. Indole is the most abundant volatile organic compound (VOC) found in control human feces (comprising between 25–90% of all VOC in feces). In patients infected with *C. difficile, C. jejuni* or active UC the indole content drops to 86, 66 and 72% of all VOC expressed in the fecal sample, respectively [61]. Other similar studies have detected both indole and IPA in blood [62] and saliva [63]. L-tryptophan feeding to humans results in increased expression of indole metabolites in blood (kyneurines, indole acetate, indole lactate) in the order of 5–10 fold from baseline values [64]. Indeed, diet can dramatically affect indole levels. In humans fed heat-stabilized rice bran (putative chemopreventive diet), there was a significant increase in microbial derived indole metabolites in the feces [65]. Another example, dietary konjac mannan fed to mice bearing human microbiota led to a reduced expression of fecal indoles [66]. In dogs fed high protein versus low protein diets, fecal indole increases [67]. On the other hand, fiber fed dogs had decreased indole production[68]. In humans, antioxidants can decrease IPA levels [69].

### 1.5. Altered tryptophan metabolism and IBD - linking microbial metabolism and host function

In humans with IBD, there is increased urinary excretion of tryptophan. Additionally, enhanced Indoleamine 2,3-dioxygenase (IDO) expression in inflamed enterocytes and rapid tryptophan catabolism in the intestines, results in low serum tryptophan but also markedly increased serum kyneurine:tryptophan ratio [70–79] and end metabolites [80, 81]. Consistent with this concept, the anti-inflammatory shunting of tryptophan to serotonin/melatonin is blunted in patients with IBD [82–84]. In addition, these observations are also consistent with the newly discovered property of IPA as an inhibitor of Human kynurenine aminotransferase I (hKATI), which catalyzes the formation of kyneuric acid, which is neurotoxic [85]. However, fecal tryptophan content is elevated which altogether suggests a block in microbial tryptophan metabolism [86]. In Hartnup’s disease, there is an almost complete lack of tryptophan absorption in vivo suggesting that the transporter involved in this disease is a major mediator of tryptophan transport. In the Hartnup fecal stream, there is elevated bacterial tryptophan metabolites, indoles and tryptamine [87]. Based on a review of epithelial amino transporters in IBD by Ghishan et al, tryptophan malabsorption and by extension of the argument, the Hartnup transporter itself (B^0^AT1/SLC6A19), has not been implicated in inflammatory bowel disease [88]. Furthermore, tryptophan deficiency (not tryptophan excess) has been implicated in colitis via the B0 AT1/ACE2/mTOR/antimicrobial transport peptide pathway [89].

These observations are also consistent with a study demonstrating that bacterially derived indoles are less abundant in patients with IBD versus healthy controls [90]. Similar results have been found in mice with radiation-mediated intestinal damage, in that, serum indole and indole metabolites (IPA) are inversely correlated with the extent of intestinal inflammation [91]. The observation that IPA is inversely related to systemic inflammation in overweight individuals [92] as well as the reversal of IPA levels (increased) upon administration of anti-inflammatory dietary interventions to humans [69] adds further proof of the importance of IPA in inflammation. Note, that obesity is a systemic disease but with evolving proof that there is substantial contribution of intestinal inflammation and dysbiosis contributing to disease pathogenesis [93–96]. Indeed, indole levels are significantly lower or absent in humans with alcohol-induced high intestinal permeability as compared to those with low intestinal permeability [97]. Additionally, environmental factors, beyond availability of tryptophan influence the coding of TnaA (induced by high pH, cell density, glucose), which are altered negatively during IBD [98–103]. In this context, enteral feeding, reduces intestinal inflammation in children with CD, and increases fecal pH [104]. Intestinal inflammation is a precursor for intestinal carcinogenesis. In this context, adenomas in humans are associated with lower indole acetate levels (IAA) in the rectal mucosa in comparison with non-adenoma controls [105]. While our work focused on IPA, we have shown that the other bacterially derived indole metabolites (e.g., IAA) also serve as PXR ligands [48].

### 1.6. Alterations in tryptophan metabolism in mouse models of intestinal inflammation

Rodent studies provide direct evidence that support a relationship between indole loss and intestinal inflammation. Mice (129S1/SvlmJ) fed westernstyle high fat diets can exhibit low tryptophan metabolism in the intestines [106]. Indeed, tryptophan metabolism is also attenuated in ApcMin/+ mice [107]. Ginseng prevents development of tumors in these mice and that is associated with induction of tryptophan metabolism [107]. Indoles chronically decrease intestinal secretion of glucagon like peptide (GLP-1) [108]. Patients with terminal ileal Crohn’s disease exhibit increased GLP-1 mRNA in enteroendocrine cells [109]. Together, these observations support a common outcome for GLP-1 expression in inflammatory states of the intestine when indole levels are reduced. Specifically, mice supplemented with L-tryptophan then exposed to inflammatory toxins (DSS, TNBS) have significantly less inflammatory indices in the intestines and less liver fat accumulation [77, 110, 111]. Mice harboring colitis have reduced indole metabolite concentrations [112]. Indeed, dextran sodium sulfate (DSS) exposed mice treated with colitogenic *Escherichia coli* have 4–7 fold lower indole levels as compared to those treated with non-colitogenic Nissle *E. coli* strain [113]. In corollary, germ-free [114] mice have high serum tryptophan compared with conventional mice [115] and when GF mice are inoculated with *E.coli* and *Faecalibacterium prauznitzii*, have increased amounts of indole-3-lactate (neither strains have the metabolic capacity to synthesize other indoles like IPA), which is associated with protection against TNBS-induced colitis [116]. In dogs with IBD, pre-treatment serum tryptophan is low when compared with normal controls [48, 117].

Overall, we estimate based on the published data that, in rodents and humans (Table 1), while fecal tryptophan levels are elevated and that indole (and in some studies, its bacterially derived metabolites) is depressed, that the tryptophan to indole ratio is likely to be greater or equal to 1. In controls without IBD, we estimate that such ratios will vary but likely be under 1. It is important to note, that in this context, diseases (that are not IBD) can have ratios greater or equal to 1, if their pathogenesis is driven by intestinal barrier/permeability defects coupled with inflammation (e.g., obesity, nonalcoholic steatohepatitis). Additionally, it is possible that enhanced epithelial absorption of IPA and consequently increased serum IPA contributes to lower fecal levels. In this context, since inflammation can lead to down-regulation of PXR, the target might not be present for effects of IPA [118]. A counter-regulatory indole response might also be a way the host reacts towards inflammation, thereby, providing a pathway for enhancing the barrier function as a means to counter the inflammatory response.

It cannot be ignored, however, that microbial metabolites of tryptophan may have concentration dependent effects on the host. For example, despite its anti-oxidant function [119], there could be deleterious effects when IPA is administered during active inflammation; however, when administered prior to active inflammation could have dose dependent protective effects in mice (unpublished data) [119]. High (non-physiologic) doses of IPA could indeed have toxic effects on intestinal organoids (unpublished data). Indeed, this could be further complicated by host metabolism (e.g., indoxyl sulphate) and such metabolites could have other consequences (e.g., uremic toxins). However, it is important to note that IPA does not accumulate in uremia and likely synthesized by a different sets of bacterial strains than indoxyl sulfate [62]. Furthermore, a recent study in patients undergoing autologous stem cell transplantation, it was concluded that low indoxyl sulfate predicted for poor outcome in these patients [120]. Thus, these observations give us pause to be cautious in performing and interpreting experiments with microbial metabolites.

### 1.7. The PXR – linking microbial metabolism and mucosal homeostasis

As outlined previously, the PXR is a target for anti-inflammatory action in the colon. Indeed, several groups had already defined some aspects of intestinal barrier regulation by PXR [34, 43, 121, 122] and our studies subsequently delineated some key mechanisms other than NF-κB signaling [123, 124]. The PXR is expressed in the colonic epithelium and we have shown an inverse relationship between PXR and TLR4 in human colonic samples obtained from health controls as well as from patients with IBD [48]. This same trend is evident in human colon cancer cell lines [48]. We have also shown that murine colonocytes exposed to a lipid A fraction (KDO2) (TLR4 agonist) in the presence of a PXR agonist (pregnane carbonitrile), results in reduced p38 phosphorylation in PXR wild-type mice but not in PXR-knockout mice (PhD Thesis, S. Mukherjee, 2013). The mechanism of TLR4 downregulation by PXR is an open and unclear area and can only be speculated as many cellular signaling components/gene expression regulation might impinge. One of the probable mechanism(s) may include modulation of kinases (necessary for signal transduction) downstream of TLR4 signaling in cells by the PXR pathway through cross-talk that would ultimately repress TLR4 activity and regulate intestinal barrier function and innate immunity. Importantly, in a collaborative study in our laboratory in *Nr1i2*^−/−^ mice (PXR-null) we observed that upon bacterial infection the downstream TLR4 pathway kinase signaling is activated with increased generation of pro-inflammatory cytokines and chemokines. What could be the mechanism(s) for this? PXR and TLR4 are both proteins and mutual regulation of their expression at the transcriptional or translational level could be a possibility. Interestingly, we found that PXR negatively regulates TLR4 signaling by reducing TLR4 mRNA stability in LS174T cells (manuscript under consideration). However, repression of TLR4 gene transcription by PXR might also be a possibility.

Then importance of the human relevance cannot be underappreciated. However, these studies have also highlighted an important fallout. If the molecular determinants of IPA (and indole) binding are delineated, then it is feasible to develop non-toxic indole based ligands based on metabolite mimicry. Furthermore, some important molecular details have been emerging that is important to review. First, PXR protein is post-translationally modified [125]. Specifically, in addition to phosphorylation, ubiquitination and SUMOylation, the receptor is acetylated [126–128]. Indeed, basal receptor activation set points could vary based on the type and degree to which the receptor is modified [128]. Thus bacterial signals apart from metabolites that induce posttranslational modifications (e.g., acetyl CoA) could affect net PXR transcriptional activity.

**Figure 2:**
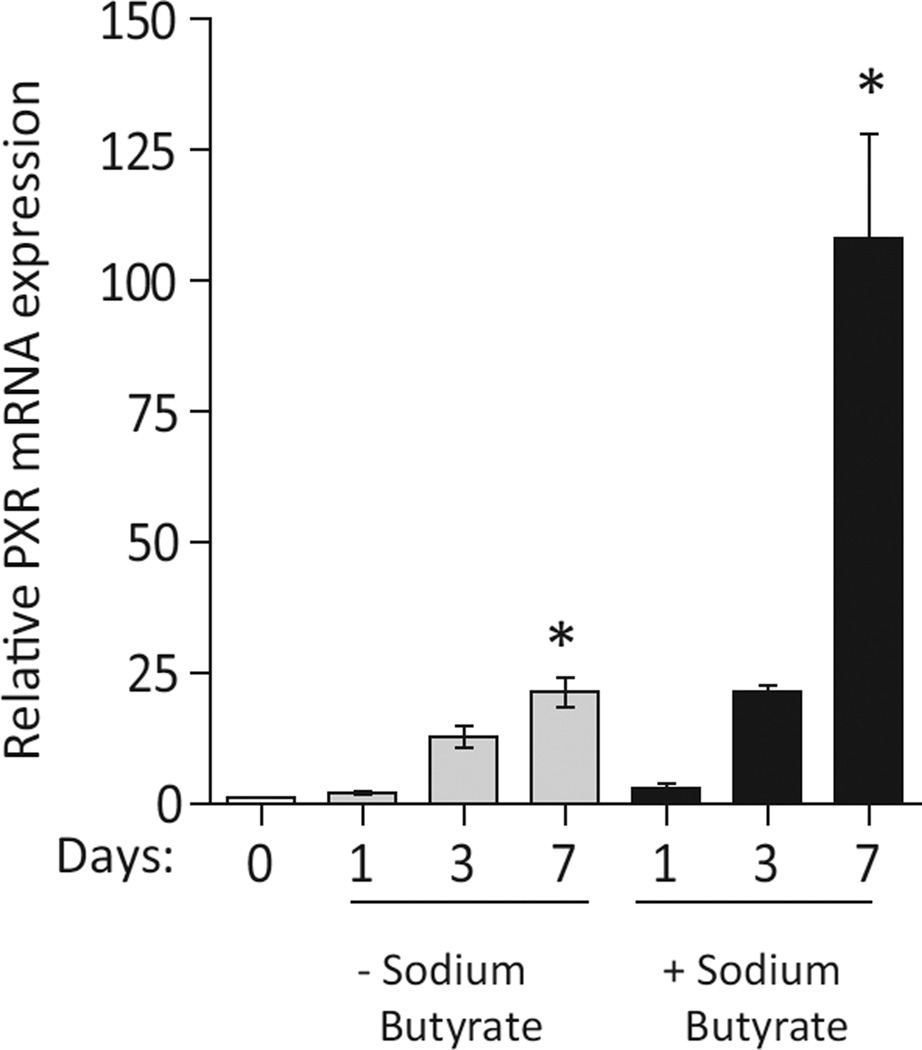
PXR mRNA expression increases in intestinal epithelial cells and murine enterocytes upon differentiation Real-time qPCR analysis of PXR expression in undifferentiated Caco-2 cells treated with the differentiating agent, sodium butyrate (1 µM). Undifferentiated Caco-2 cells were allowed to differentiate for 7-days post-confluence, in the presence of sodium butyrate. On analysis we observed that PXR mRNA levels increased several fold upon sodium butyrate-induced differentiation. Gene expression changes were calculated using the comparative C_*t*_ method with β-actin and GAPDH as the reference genes. Data expressed as fold change in mRNA expression compared to undifferentiated cells. Experiment was performed at least two times in triplicate. Graph show mean values ± s.e.m. *P ≤ 0.05 (One-way ANOVA) [212].

Another intriguing aspect relevant to the intestine, is the concept of shear forces that are applied to the intestinal villi during peristalsis and food movement. Recently, it has been shown that mechanical shear forces induce endothelial PXR transcription activation [129]. We have also shown that short chain fatty acids, in particular butyrate, induces PXR transcription in Caco-2 cells (Figure 2) (PhD Thesis, S. Mukherjee, 2013). So, our model presents a framework for the regulation of the intestinal barrier via PXR but it also suggests dynamicity in its regulation that is affected by multiple factors. One strong hypothesis, but which needs to be proven by experiments is that the indole sensing pathway might get negatively regulated in the presence of “transient pathogens” or “indole-metabolizing bacteria”, which would result (based on our model) in a more permeable intestinal barrier due to loss of PXR signaling. This could result in increased luminal sensing of these pathogens by sub-mucosal lymphoid cells and resulting inflammatory response could act to prevent invasion by these species. Transient pathogens are present in the normal flora and are likely kept at bay via such (similar) mechanisms. This is a physiologic response ongoing in “normal flora” and perhaps a host mechanism to buttress immune homeostasis via a “transient inflammatory” state largely characterized by heightened cytokine expression (e.g., TNFα). Indeed, as we progress forward towards clinical translation, these factors are crucial to control for in order to reduce noise and misplaced conclusions.

### 1.8. The Aryl Hydrocarbon Receptor – an additional link between microbial metabolism and the regulation of intestinal mucosal homeostasis

In addition to the PXR, the aryl hydrocarbon receptor (AhR) plays a key role in sensing and responding to microbial metabolites in the intestinal mucosa and has been reported to play an important role in maintaining intestinal epithelial barrier function and mucosal homeostasis. The AhR is a cytosolic transcription factor of the basic helix-loop-helix Per/Arnt/Sim (bHLH/PAS) receptor family [130], whose activity is tightly regulated through interactions with a number of chaperone proteins and actin filaments, keeping the receptor sequestered until it encounters high-affinity ligands. Binding of ligands to AhR prompts its translocation to the nucleus where it pairs with aryl hydrocarbon receptor nuclear translocator (Arnt) forming a complex that interacts with dioxin response elements (DRE) present in AhR target genes. These genes are involved in a number of processes ranging from xenobiotic sensing to environmental adaptations [130, 131]. Several ligands of physiological relevance have been identified for the AhR, with most being the product of tryptophan metabolism. The major pathway for tryptophan metabolism in the body produces kynurenine that can bind AhR, but lacks potency [131]. Therefore, focus has been placed on AhR ligands derived from environmental sources. These include dietary intake that can be catabolized into AhR ligands. For example, the breakdown of glucobrassicin in cruciferous vegetables such a broccoli or cabbage produces indole-3-carbinol (I3C). Under acidic conditions in the stomach I3C is broken down into indolo-[3,2-b]-carbazole (ICZ) and 3,3’-diindolylmethane (DIM), both of which have been identified as high affinity AhR ligands. Like the PXR, bacterial metabolites can also bind to and promote signaling via the AhR. Indoles can be generated from bacterial metabolism of tryptophan, which is a major metabolic pathway in certain bacteria. Several bacteria that can produce indoles from tryptophan have been reported, including the members of the intestinal microbiota *Lactobacillus reuteri* [132], *L. helviticus* [133], and *Clostridium sporogenes* [50]. Interestingly, there is selectivity towards indoles that activate AhR - IPA does not activate AhR [48, 134, 135]. Another physiological ligand for AhR produced in the body that is worth noting is FICZ, which results from photolysis of L-trytophan upon exposure to both visible and UV light. FICZ is found naturally in the skin [136] but has also been utilized to activate AhR activity in different organ systems including the intestine [137].

The AhR is expressed throughout the body including barrier surfaces such as the skin, lung and intestine. The role of AhR is of particular interest in the intestinal mucosa given its interactions with environmental, dietary and bacterial derived ligands. AhR, is expressed in the intestinal epithelium [138] as well as by a number of intestinal immune cell types, especially those at the luminal interface where interactions with ligands and antigens are frequent [131]. In the intestine, AhR activation has shown to be protective in different models of intestinal inflammation [132, 137, 139–141]. Interestingly, tissue from patients with IBD expressed significantly less AhR than controls [137]. The protection allotted by AhR signaling occurs through a number of mechanisms linked to its ability to influence different immune responses, particularly those involved in intestinal barrier function. One cytokine central to the ability of AhR to promote mucosal defense and to promote barrier function is interleukin (IL)-22 [137]. IL-22 targets the epithelium where it increases epithelial cell proliferation, cell migration, and mucus production [142, 143]. IL-22 also induces the expression of several antimicrobial peptides that have direct microbicidal effects [144, 145]. These combined effects of IL-22 are important in containing luminal bacteria while also repairing compromises in the intestinal barrier. IL-17A expression is also influenced by AhR activation where it can promote the function of innate mechanisms [146] and augment antimicrobial peptide expression induced by IL-22 [147].

IL-22 and IL-17A are signature cytokines of Th17 cells, which have also been shown to express high levels of the AhR. Th17 cells can be found in the intestinal lamina propria throughout the small and large intestine where they are important for controlling extracellular pathogens but can also promote pathogenic responses [146]. AhR expression on Th17 cell augments their development and differentiation both *in vitro* and *in vivo* [148–150]. Interestingly, AhR^−/−^mice actually harbor increased numbers of Th17 cells in the intestine. Further investigation into these mice revealed increased colonization of the gut with segmented filamentous bacteria (SFB) as a result of defective IL-22 expression. The presence of SFB in the gut is a potent stimulator of Th17 differentiation and thus, increased numbers of Th17 cells are the result of the inability of IL-22 to control SFB levels. Another subset of intestinal T cells termed Th22 cells, which are characterized for their ability to produce IL-22 but not IL-17A, also rely on AhR for development. IL-22 produced by AhR expressing Th22 cells was critical for protection against infection with the extracellular pathogen *C. rodentium* [151]. A similar subset of T cells dependent on AhR and produce IL-22 but not IL-17 has been identified in humans [152]. Finally, recent evidence also established a novel role for AhR in the plasticity of intestinal CD4^+^ T-cell responses during inflammation [153]. Specifically, it has been reported that pathogenic Th17 cells can transdifferentiate into anti-inflammatory type 1 regulatory T cells (Tr1 cells) during the resolution of colitis. The trans-differentiation process, which lends to the quelling of intestinal inflammation occurs in the presence of TGF-beta and requires AhR signaling [153].

AhR is also expressed by RORyt+ innate lymphoid cells (ILCs), which are a major source of IL-22 at the intestinal barrier [154, 155]. RORyt+ ILCs from AhR^−/−^ mice exhibit increased apoptosis and decreased production of IL-22. In agreement, AhR^−/−^ mice are more susceptible to *Citrobacter rodentium* infection [141]. Additionally, AhR^−/−^ mice also display expansion of the adherent bacteria SFB, which, in turn can promote the expansion of pathogenic Th17 cells, which can fuel intestinal inflammation. The ability of AhR-expressing ILCs to ameliorate *C. rodentium* infection has also been linked to the ability of IL-22 derived from ILCs to maintain a proper microbiota that aids resisting the colonization of pathogens [139]. In these situations, AhR ligands appear to be derived from either the microbiota or dietary intake. Indeed, microbiota-derived AhR ligands have been shown to stimulate ILCs to influence intestinal barrier function and prevent mucosal inflammation. In one study, Lactobacillus reuteri, a commensal bacterium, metabolized tryptophan via aromatic amino acid aminotransferase to produce the AhR ligand indole-3-aldehyde [132]. Ligation of AhR in ILCs with the microbial metabolite increased levels of IL-17A and IL-22, which aided in the resistance to colonization with *Candida albicans* and prevented mucosal inflammation. Alternatively, dietary AhR ligands were able to stimulate organogenesis of intestinal lymphoid follicles through their activation of AhR-expressing ILCs [156]. Interestingly, AhR activation is also required to maintain population of intraepithelial lymphocytes (IELs), which reside in the intestinal epithelium and are in direct proximity to the intestinal lumen and its contents. AhR^−/−^ mice or mice fed a synthetic diet lacking phytochemicals that can engage the AhR were shown to lack IELs. In both cases mice also displayed decreased epithelial proliferation, increased bacterial growth, increased immune activation and were more susceptible to colitis induced by DSS [140].

Microbiota-derived metabolites acting as ligands of intestinal AhR could serve as an effective mechanism in the control of gut inflammation and physiology. Indole 3-acetate, tryptamine and indole are tryptophan-derived gut microbial metabolites that has been shown to modulate AhR activity in Caco-2 human epithelial colon cancer cells and other cells [157]. Mice cecal and fecal samples when analyzed showed the presence of above three metabolites in modest concentrations. *In vitro* study in AhR-expressing Caco-2 cells showed strong agonist activity of indole 3-acetate and tryptamine as observed by increased expression of AhR-responsive genes such as Cyp1A1 and Cyp1B1 mRNA and protein when compared to AhR standard agonist TCDD (2,3,7,8-tetrachlorodibenzo-p-dioxin) [157]. However, indole repressed AhR activity by acting as an antagonist. Interestingly, the metabolites exhibited low anti-inflammatory activities, whereas TCDD had AhR-dependent anti-inflammatory effect. In a recent report the above tryptophan metabolites including indole 3-aldehyde were studied as modulators of AhR activity in non-transformed young adult mouse colonocytes [158]. AhR-dependent induction of Cyp1A1 mRNA by these metabolites show weak agonist and partial antagonist activities with an altered activity pattern contrary to what was previously seen in Caco-2 cells. Using other AhR-responsive genes like Cyp1B1, the AhR repressor (Ahrr), and TCDD-inducible poly (ADP-ribose) polymerase (TiParp) showed complicated pattern of AhR agonist and antagonist activities of these metabolites that were both ligand and gene-dependent thereby acting as selective AhR modulators [158]. This shows that tryptophan metabolites by modulating AhR transcriptional regulation might be selective and specific players in the control of intestinal epithelial cells interaction with the microbiota in the luminal environment.

Together these studies establish physiological functions for AhR in the gut. AhR is required to resist intestinal inflammation in a variety of models, while also promoting proper barrier function. Importantly, clear evidence shows that AhR activity, much like other nuclear receptors can be modulated by both dietary and bacterial metabolites. It must be noted that the responses mediated through AhR have profound effects on the microbiota and host function. At the same time there may be significant interplay between diet and its effect on tryptophan-metabolizing bacteria and ultimately, nuclear receptor signaling in the intestine. This represents an intriguing area for future study.

## 2. Other Host Nuclear Receptors - Bridging With the Symbiotic Microbiota

As discussed above, it points with little doubt that at present PXR and AhR are known to be the prime xenobiotic NRs that take part in luminal xenobiotic sensing and intestinal-microbiota relationship. This is true as majority of the reports link these two receptors in such functions. However, over the years, many other members of the NRs superfamily have also made inroads into this area and shown to be participants in gut microbiota metabolism and mucosal homeostasis. In this section, new and recent developments in gut resident microbiota *vis a vis* some other NR members shall be reviewed. The PPARγ (NR1C3) is a well-known and important member of a subfamily (NR1C) belonging to the NRs superfamily. PPARγ is activated by fatty acids as ligands with major roles in glucose and lipid metabolism and is implicated in host insulin resistance, hyperlipidemia and other functions [159]. PPARγ is highly expressed in the white and brown adipose tissues, and at low levels in other organs including the intestine. It is reported that microbial-derived propionic acid (PA) in the gut promote immuno-suppressive effects and improve insulin sensitivity in the host [160]. One of the modes of actions of PA in the host includes binding and activation of PPARγ, which in turn inhibit NF-κB-mediated gene transcription activity and modulating inflammatory signaling pathways in the intestinal tract [160]. Also, PPARγ has been demonstrated to possess anti-inflammatory effect in experimental IBD. The expression of PPARγ gene itself in the intestinal epithelial organoids culture is activated by the abundant gut bacterium *Akkermansia muciniphila* secreted propionate and might be true in intestinal epithelial cells also which may have significant gut metabolic consequences [161]. Circulating factors play important role in metabolic homeostasis by communicating with the microbiota and the intestine. A report shows that one such factor is a protein called angiopoietin-like 4 (ANGPTL4) [162]. ANGPTL4 is secreted by multiple tissues/organs including the intestine and regulate the level of plasma lipids and its overexpression enhances plasma triacylglycerol level [163]. The expression of ANGPTL4 is under the control of PPARγ in human colon. Microbial-secreted acetic acid, PA and butyric acid acting as ligands activate PPARγ and increases the expression of ANGPTL4 in human colon adenocarcinoma cells [162]. However, in another study, butyrate increased intestinal ANGPTL4 expression in human IECs independent of PPARγ signaling and that co-treatment with PPARγ ligand rosiglitazone and butyrate increased ANGPTL4 expression in an additive manner [164]. This indicates that PPARγ and butyrate drive ANGPTL4 expression through two different pathways or gene regulatory elements. Indeed, it was revealed that butyrate signaling unlike PPARγ was through a distinct GC-rich sequence upstream of start site in the ANGPTL4 gene promoter. However, acetate and propionate action on ANGPTL4 expression was largely PPARγ-dependent. Microbiota-derived SCFAs thus act as novel modulators of PPARγ in colonic cells and may contribute towards host circulating lipid levels.

In cultured enterocytes, a range of microbiota including *E. coli*, Bifidobacteria, Lactobacilli, Propionibacterium, Bacillus and Saccharomyces modulate the expression of intestinal TLRs, mucins, caspases, interleukins and NF-κB which have important anti-inflammatory consequences [165]. Moreover, interaction of the microbial population with the surface antigens of antigen presenting cells (APCs) *in vitro* is shown to down-regulate and up-regulate the expression of various pro-inflammatory and anti-inflammatory genes, respectively [165]. Collectively, microbiota-mediated effects on the immune response help in controlling growth and invasion of potential pathogens in the host. Some NRs may have a role in modulating the immune response during host-microbiota interactions. The retinoic acid receptor (RAR) when activated by its cognate endogenous ligand all-*trans* retinoic acid (RA) maintain immune homeostasis in the intestine. RA availability in the intestine is dependent upon gut microbial action on ingested dietary vitamin A (retinol). RAR-mediated control of gene expression in the intestine is necessary to achieve immune tolerance towards dietary antigens [166]. Such immune tolerance might be helpful in understanding the causes of food allergies, including IBD.

Farnesoid X receptor (FXR) is expressed in multiple tissues/organs, with high levels in the intestine and functions in regulating carbohydrate and lipid metabolism. FXR is activated by bile acids and appears to have anti-inflammatory functions in experimental models of IBD. Conjugated bile acids are released by the liver into the intestine where they are converted to secondary unconjugated bile acids by the microbiota which are more toxic and can cause pathological complications [167]. In the intestine, FXR activation by bile acids protects against microbiota overgrowth and composition and facilitate anti-inflammatory actions. Obstruction of bile acids flow to the intestine facilitate bacterial overgrowth and injury to the mucosa. The host bile acids in the gut undergo chemical modifications by the resident microbiota to generate bio-transformed bile acids that modulate FXR signaling which have important consequences in controlling microbial composition and mucosal inflammation [168]. In germ-free mice, conjugated tauro-β-muricholic acid (T-β-MCA) exhibit antagonistic property against FXR [169]. Inhibition of intestinal *Lactobacilli* and reversed *Firmicutes/Bacteroides* ratio upon treatment with tempol (an anti-oxidant that reduces obesity in mice) led to increased T-β-MCA accumulation that inhibited intestinal FXR activity leading to decreased obesity in mice [170]. Tempol treatment did not reduce obesity in FXR-null mice indicating that intestinal FXR signaling contributed to the reduced obesity effect of tempol suggesting biochemical cross-talk between the microbiota, FXR and metabolic disorders such as obesity.

Bile acids thus appear to be relevant metabolites that can influence intestinal microflora and physiology [171]. Aberration in bile acids metabolism in the gut linked to microbiota composition may be relevant in nonalcoholic hepatic steatosis. A recent study in Ob/ob mice showed that butyrate-synthesizing bacteria in the gut is considerably lower in proportion along with reduced fecal taurine-conjugated bile acid than their lean counterparts which stimulates nonalcoholic hepatic steatosis [172]. On the other hand, intestinal expression of FXR primary transcript and hepatic Cyp7A1 and SHP were upregulated and downregulated respectively in Ob/ob mice with respect to controls. This suggests that gut bacteria-dependent bile acid deconjugation in Ob/ob mice activates FXR activity and inhibit the FXR-small heterodimer partner (SHP) signaling pathway leading to increased fat biosynthesis and nonalcoholic hepatic steatosis. This provides a strong evidence that microbiota composition and their metabolites in the gut can influence bile acid metabolism and potentiate non-alcoholic hepatic steatosis [171].

FXR is expressed by the immune cells in the intestinal tract and modulate innate immunity and is shown to regulate inflammation in animal models of colitis. LPS-activated macrophages when treated with 6-ethyl chenodeoxycholic acid (INT-747), a FXR agonist, repress the expression of NF-κB regulated genes such as TNF-α, IL-1beta, IL-6, COX-1, COX-2 by a mechanism involving stabilization of NCoR on the NF-κB responsive element of these genes [173]. Moreover, in inflamed colon of Crohn’s patients, decreased FXR mRNA expression is observed. FXR^−/−^mice when subjected to colitis showed increased inflammatory condition and treatment with INT-747 reduces innate immune cell activation accompanied with upregulated expression of FXR and SHP while attenuating the expression of primary transcripts of IL-1beta, IL-6, TNF-α and IFN-γ [173]. TLRs are crucial in sensing the intestinal microbiota and play a role in innate immune response [174]. Evidences indicate that gut microbiota modulate the expression of few NRs in the GIT which have implications on the overall well-being of the gut including maturation of the host immune system [175, 176]. Dysregulated FXR expression has a role in intestinal inflammation. It is visualized that the expression of FXR can be regulated by the TLRs [176]. In fact, the expression of FXR but not the other NRs is positively up-regulated by TLR9 signaling in the intestine and that FXR contributes to the cellular activities governed by TLR9 [177]. Studies have demonstrated that FXR activation has a positive influence on intestinal barrier structures and functions including a protective role in IBD mediated via antagonizing the TLR 4/TNFα and NF-κB signaling in mice colonic mucosa and several types of immune cells [178–180]. Finding suitable, non-toxic agonists of FXR may be helpful in treating IBD.

The VDR is another NR that has numerous impact on physiology and its downregulation is associated with several diseases such as IBD, obesity, diabetes, cancers and asthma. VDR is thought to regulate microbiota composition in the gut through little known mechanism(s). Interestingly, VDR^−/−^mice fecal samples exhibited altered gut microbiota profile with Lactobacillus depletion, accompanied with Clostridium and Bacteroides enrichment [181]. Also, VDR KO mice show increased IL-22 producing innate lymphoid cells and antibacterial peptides than the wild type counterparts[182]. This indicates that vitamin D acting through VDR is vital to maintenance of gut microbiota and GIT homeostasis. Preliminary studies show VDR as a regulator of intestinal permeability. Human subjects and animal models with IBD show defective intestinal barrier functions and increased permeability. VDR KO mice subjected to dextran sodium sulfate (DSS)-induced colitis failed to maintain the integrity of intestinal barrier (due to reduced junctional proteins) accompanied with increased gut permeability compared to the wild type which could be linked to the altered gut microbiota composition [183, 184]. VDR KO mice are highly susceptible to experimental IBD associated with altered gut microbiota population. However, antibiotic treatment of VDR KO mice provided protection towards IBD symptoms [185]. VDR upon activation by vitamin D also regulate innate immunity towards gut microbiota by controlling the expression of several pattern-recognition receptors such as Nod2 in the gut epithelial cells [186, 187]. The effect of VDR on microbiota is indirect as it is the host which expresses the receptor and therefore VDR-mediated changes in the host ultimately modulate the microbiome that enable in the maintenance of immune tolerance in the gut.

The retinoid-related orphan receptors (RORs) are a group of NRs with three members (RORα-γ) exhibiting distinct tissue-specific expression pattern. However, using alternative splicing, multiple isoforms of RORs are known that vary in amino acid sequence at the N-terminus [188]. RORs control numerous biological functions including immunity. The role of microbiota in regulating gut immune system functioning and development is confirmed and in turn the intestinal immune cells regulate microbiota composition and functions. A large population of intestinal T_*reg*_ cells expresses RORγt and the presence of active gut microbiota is necessary towards induction and maintenance of RORγt-expressing T_*reg*_ cells. T_*reg*_ population is significantly reduced in germ-free mice, however, introduction of microbiota facilitates RORγt-expressing T_*reg*_ cells [189, 190]. Intestinal RORγt expression in T_*reg*_ cells was shown to be regulated by the microbial metabolite butyrate and retinoic acid which can have important consequences in intestinal innate immunity [189, 190]. In the intestine, the microbiota by inducing IL-7 expression stabilizes the expression of RORγt^+^ in intestinal lymphoid cells (ILCs) that plays a role in intestinal lymphoid follicles development [191]. Interestingly, lack of microbiota-mediated IL-7 induction facilitate differentiation of RORγt^+^ ILCs into RORγt^−^ IFN-γ–producing pathogenic ILCs that facilitate intestinal inflammation. RORα in the intestinal epithelia regulate many microbe sensing receptors including Nod2 and TLRs [192]. In RORα KO mice expression of these genes are downregulated. Interestingly, in microbiota-depleted mice, intestinal RORα expression is downregulated which points to the existence of regulatory axis between gut microbiota, innate immunity genes and RORα [192].

Microbiota in the GIT are under immense pressure from the host dietary intake and food patterns which affect their composition, diversity and secreted metabolites. Differences in secreted metabolites in the gut such as butyrate and folate depend upon nutrition and microflora composition and diversity. It is proposed that these metabolites can exert epigenetic modifications in the intestinal epithelial cells epigenome that can have significant effects on microbiota-gut relationship [193]. More specifically, epigenetic changes (DNA methylation or histone modifications) might be induced in genes coding NRs in the intestine such as RXR, LXR that have may modulate signaling with the microbiota [194, 195]. LXR is an important NR in controlling lipid metabolism and particularly cholesterol balance in the body. In the gut, microbial action on the host-dietary choline, phosphatidylcholine and carnitine is important. Dietary choline and L-carnitine are converted to trimethylamine (TMA) by a gut bacterial pathway which then migrates to the liver where it is oxidized to TMA N-oxide (TMAO) by the hepatic flavin monooxygenase 3 (FMO3) [196]. TMAO has pro-atherogenic effect and is being linked to cardiovascular risk in humans [197] and a recent study correlates TMAO with increased platelet responsiveness and activation which may fuel the risk of thrombosis in the cardiovascular system [198]. TMAO in human subjects stimulated platelet adhesion to collagen of endothelial cells of the blood vessels. Injection of TMAO to mice enhanced thrombus development in the internal carotid artery by a mechanism involving increased stimulus-dependent release of stored intracellular calcium in platelets leading to elevated platelet activation and responsiveness [198]. Interestingly, germ-free mice supplemented with choline did not produce TMA and hepatic TMAO and subsequently no thrombosis. Moreover, in a metagenomic study, eight bacterial genera associated with the production of TMA were identified in mice such as *Anaerococcus hydrogenalis, Clostridium asparagiforme, Clostridium sporagenes, Escherichia fergusonii* and others [199]. Hepatic FMO3 is also associated with modification of cholesterol metabolism and reverse cholesterol transport [200]. Hepatic FMO3 expression is induced by dietary bile acids which is LXR-dependent [201]. Mice with FMO3 knock-down and fed with cholesterol exhibit altered cholesterol balance, low hepatic oxysterols and high endoplasmic reticulum (ER) stress and increased inflammation. This results in limited oxysterols which leads to low LXR activation. However, FMO3 knock-down activates LXR-mediated macrophage reverse cholesterol transport pathway [200]. FMO3 is thus seen as a player in unifying cholesterol balance, inflammation and ER stress. Thus TMA/TMAO/FMO3 and LXR signaling seems to be an important regulator of cardiovascular system, cholesterol metabolism and inflammation.

Polyaromatic hydrocarbons (PAHs) are widely used commercial chemicals and are present in many materials, including food and drink. PAHs are not estrogenic and use of pure naphthalene, phenanthrene, pyrene, and benzo(*a*)pyrene up to 16 µM did not show any estrogenic potency in estrogen bioassay [202]. Small intestine digests of these PAHs also did not reveal any estrogenicity, however, gut PAH digests showed significant estrogenic response in *in vitro* study. Heat-inactivated colonic suspension showed drastic decrease in estrogenic response indicating role of gut microbiota in PAH biotransformation [202]. Moreover, using PAHs at low and in human relevant concentrations showed modest estrogenic activities from colon digests. Microbial-hydroxylated PAHs resemble natural estrogens in structure and hypothetically through the circulation may ultimately bind and activate estrogen receptors (ERs) in different organs/tissue causing potential health risks. However, it needs to be tested whether PAH biotransformation by the gut microbiota actually occurs in the GIT of intact animals.

Therapeutic drugs (xenobiotics) in human are primarily metabolized in the liver. Host microbiota and the metabolism of xenobiotics appear to be intricately linked via modulating the expression of drug-metabolizing enzymes [203]. PXR and constitutive androstane receptor (CAR) control the expression of hepatic drug-metabolizing genes. Intestinal microbiota influences hepatic gene expression of PXR and CAR which influence xenobiotic metabolism. Using oligo microarray and qPCR approaches, it was demonstrated that the gut microbiota modulate the expression of several hepatic genes, many of which metabolize xenobiotics through the activation of CAR [204]. Hepatic CAR expression was elevated in germ-free mice compared to specific pathogen-free mice with differential expression of CAR-regulated genes. The expression of CAR-regulated hepatic cytochrome P450 (Cyp 450) oxidoreductase was upregulated in germ-free mice as against specific pathogen-free mice. The upregulation of CAR and Cyp 450 in germ free mice indicate microbial influence of gene expression in the hepatocytes. Presence of active microbiota limits expression of CAR and Cyp P450 oxidoreductase which may decrease drug biotransformation and detoxification thereby increasing the bioavailability of the drug to the host. Germ-free mice are an excellent model to study the effect of gut microbiota on host drug metabolism. PPARα and PXR regulate the expression of specific hepatic cytochrome P450’s genes *Cyp4A* and *Cyp3A* respectively. The expression of certain *Cyp3A* and *Cyp4A* maybe modulated in germ-free mice. In fact, the expression of PXR primary transcript was upregulated in germ free mice compared to conventional mice however, with concomitant decrease in the microsomal activities of hepatic Cyp3a11 and Cyp3a44 enzymes [205]. PPARα expression in germ-free mice was upregulated with significant increase in the hepatic expression of cyp4A10 and cyp4A14 transcript and protein [206]. These study, including many others reflect a clear and present role of gut microbiota in modulating hepatic drug metabolism whereby one of the routes that are exploited is by regulating hepatic CAR and PXR expression.

## 3. Concluding Statements

The purpose of this review is to summarize new developments in the interface of microbial metabolites and host mechanism(s). Having said this, there is a panoply of NRs (e.g., FXR, PPARγ, RORγt) [207–210], expressed in the intestinal epithelium as well as innate immune cells, that exert a major influence on the intestinal barrier function and innate immunity. However, our review primarily summarizes recent advances in two very related NRs – one (PXR) that is predominantly expressed in the intestinal epithelial cell while the other (AhR) in the immune and epithelial compartment. Both receptors participate in “epimmunome” signaling [211], in that, they instruct complementary immune cells via epithelial signaling to regulate innate immunity. Moreover, we have also reviewed the role played by few other NRs that are increasingly seen to be part of the gut microflora, intestinal barrier and innate immunity axis. The purpose of our review was to introduce strong conjecture of possible ways further research may be posed in answering vexing questions on the interface between nuclear receptors, microbial metabolite signaling and host immunity. To do this we have kept the title broad, but focused intensely on two very similar but genetically distinct receptor systems that have complementary effects on intestinal immunity.

## Figures and Tables

**Table 1:** Indole microbial metabolites and correlation with intestinal barrier driven disease states [213].

Metabolite	Compartment	Disease	Correlation	Reference(s)
Indole	Feces	ulcerative colitis	decreases ∼ 28%	[61]
Indole	Blood	alcohol dependenceΦ	see notes below	[97]
Indoxyl sulfate*	Urine	acute GI GVHD	decreases ∼ 92%	[214]
Tryptophan	Serum	IBS	elevated ∼ 5-fold	[215]
Indole propionate	Blood	Obesity	decreased pre-treatment	
Indole propionate	Blood	Obesity	increased post-treatment**	[92]

* Compare this to expression of Kynurenine, which are significantly increased in acute graft versus host disease (GVHD) with gastrointestinal (GI) symptoms [216].

° Kynurenine is also significantly elevated downstream of Toll-like Receptor (TLR) activation in patients with Irritable bowel syndrome (IBS) [217, 218].

Φ indole levels are lower or absent in alcohol dependent patients with high intestinal permeability as compared with alcohol dependent patients with low permeability

** treatment refers to antioxidants to reduce obesity related systemic inflammation
